# The Biopsychosocial Impact of COVID-19 on Older Adults

**DOI:** 10.1177/23337214211034274

**Published:** 2021-08-13

**Authors:** Nazeefah Laher, Sara Bocchinfuso, Madeline Chidiac, Claire Doherty, Alexandra Persson, Emma Warren

**Affiliations:** 1 155276 Trinity College Dublin School of Medicine, Dublin, Ireland

**Keywords:** COVID-19, SARS-Cov-2, biopsychosocial model, older adults

## Abstract

COVID-19 has spread rapidly around the world and taken over 2.6 million lives. Older adults experience disproportionate morbidity and mortality from the disease because increasing age and the presence of comorbidities are important predictors of negative outcomes. Lasting effects of COVID-19 have been described after recovery from the acute illness despite eradication of the virus from the body. The impact of COVID-19 on a person’s biological health post-infection is observed in multiple systems including respiratory, cardiac, renal, haematological, and neurological. Psychological dysfunction following recovery is also prevalent. Social factors such as distancing and stay at home measures leave older adults isolated and food insecure; they also face intertwined financial and health risks due to the resulting economic shutdown. This study examines the effects of COVID-19 on older adults using the biopsychosocial model framework.

## Introduction

Severe acute respiratory syndrome coronavirus 2 (SARS-CoV-2), the virus that causes coronavirus disease (COVID-19), was first discovered in Wuhan, China in December 2019 (Shahid et al., 2020). By 11 March 2020, the World Health Organization (WHO) declared COVID-19 a global pandemic. As of 11 March 2021, there have been 118,343,420 global cases and 2,625,729 global deaths (Johns Hopkins University, 2021).

Age poses a significant risk of mortality due to COVID-19. In the United States (US), eight out of every 10 deaths from COVID-19 are reported in people over 65 years of age (Centre for Disease Control and Prevention, 2020b). Wiersinga et al. (2020) report a mortality rate of 0.3 deaths per 1000 among 5- to 17-year-olds, in contrast to 304.9 deaths per 1000 seen amongst those over 85. Furthermore, an analysis conducted by WHO–China has found that patients at the highest risk of severe disease and death are those over the age of 60 with comorbidities (Shahid et al., 2020). Comorbidities associated with worse outcomes are cardiovascular disease, diabetes mellitus, chronic pulmonary disease, chronic kidney disease, and chronic liver disease (Docherty et al., 2020).

Categorical definitions of old age are not only difficult to quantify, but are often not universally acceptable as calendar age and biological age are not synonymous across populations. For the purposes of this study, the term older adult is used to describe individuals aged 65 years or older. The definition is arbitrary, but it is in line with both the WHO categorization, wherein older adults are aged 65+ (WHO, 2001), and also the formal retirement age in many Western countries. However, it must be noted that studies published globally do have slight age deviations and differ in their definition of older adults and their categorization of COVID-19 rates by age.

The biopsychosocial model explores the interconnection of psychological, social and biological mechanisms as the determinants of health and disease. This model was first proposed by George Engel in 1977 and changed the way we understand and conceptualize a disease (Engel, 1977). Engle states that ‘the boundaries between health and disease, between well and sick, are far from clear and never will be clear, for they are diffused by cultural, social, and psychological considerations’. This model emphasizes that a person’s physical state is heavily dependent on their social and mental state, and these elements of disease must be considered together rather than as separate entities. This is especially relevant to the COVID-19 pandemic, as it acknowledges the relationship between biological, psychological, and social status among patients (Ali Jadoo, 2020). Individually, each component is insufficient to encompass the impact of illness and disease on a person’s health. Instead, it is the deep interrelation of all three factors that has to be considered.

COVID-19 disproportionately affects older adults. This study aims to look at the impact of COVID-19 on older adult survivors through the lens of the biopsychosocial model.

## Search Strategy and Study Selection

As part of the search strategy and study selection, a series of steps were undertaken. First, the research question was identified, “How can the biopsychosocial model be applied to understanding the impact of COVID-19 on older adults?”. A search strategy was then developed to identify peer-reviewed academic literature and relevant grey literature that supported the research question. As part of the search strategy, inclusion criteria consisted of peer-reviewed studies published in the last 20 years (from 2001 onwards) in English.

Included studies supporting the application of the biopsychosocial model on COVID-19 in older adults had to involve older adults in their study population. While this study defines older adults as individuals 65 years of age and older, based on the WHO definition (WHO, 2001), global studies vary in their age deviations and differ in their definition of older adults. For this reason, the inclusion criteria of older adults were expanded to include peer-reviewed articles that studied older adults, irrespective of how studies defined older adults by age. Given the emerging data surrounding COVID-19, it was reasonable not to exclude studies based on global variations in the definition of older adults.

Grey literature selected was used to obtain universal definitions, such as from the WHO, or to support peer-reviewed data, such as from the Centre for Disease Control and Prevention (CDC). Grey literature selected also had to meet the same inclusion criteria as highlighted above and had to come from reputable, internationally renowned sources.

Databases used to search for relevant literature included Google Scholar and PubMed, and searches included keywords such as ‘COVID-19’, ‘SARS-Cov-2’, ‘older adults’, ‘biopsychosocial’, ‘mental health’, ‘social isolation’ and ‘health’.

Both quantitative and qualitative data were obtained from all published literature that met the search criteria, allowing the authors to build a broad and comprehensive framework for understanding how COVID-19 impacts the older adult population. Concepts were organized thematically into biological, psychological, and social components of health.

## Biological

COVID-19 generally presents as an acute but mild respiratory syndrome in most patients. However, as mentioned above, it has proven to be a significant cause of mortality and fulminant disease in older survivors (Wu & McGoogan, 2020).

Angiotensin-converting enzyme (ACE) 2 receptors have been implicated as the functional receptor leading to COVID-19’s manifestation as primarily a respiratory illness with a continuum of additional extrapulmonary complications (Bourgonje et al., 2020). The active and post-infective stage of COVID-19 is often characterized by multi-organ dysfunction. Conditions such as acute respiratory distress syndrome (ARDS), acute kidney injury (AKI), cardiovascular complications including thromboembolic events, and neurological sequelae have significantly challenged disease management (Zheng et al., 2020). The development of viral sepsis caused by a dysregulated host response contributes further to critical manifestations of COVID-19. As such, the sequelae seen following the above conditions, including new physical disability and significant health deterioration, are likely to be seen in COVID-19 survivors (Cecconi et al., 2018).

### Respiratory Manifestations

To date, pathological findings at autopsy have resembled those previously seen in severe acute respiratory syndrome (SARS) and Middle East respiratory syndrome, whereby diffuse alveolar damage has been the most characteristic finding (Xu et al., 2020). Manifesting clinically as ARDS, this has been the most critical pulmonary complication precipitating the high rate of mortality and is associated with admission to the intensive care unit, mechanical ventilation and death (Shi et al., 2020). Moreover, ARDS is inherently connected to a high prevalence of cognitive impairment, decreased functional ability, and quality of life after discharge (Sasannejad et al., 2019). In addition, new onset illness, fatigue, and breathlessness are reported in over 60% of patients with COVID-19 discharged from hospital (Halpin et al., 2020). Impaired diffusion capacity, restrictive ventilatory defects, and reduced exercise capacity plague survivors post-discharge and have been associated with disease severity (Mo et al., 2020).

### Cardiac Manifestations

Putative causes of acute myocardial injury linked to COVID-19 have included myocarditis, cardiomyopathy, and ventricular arrhythmias (Akhmerov & Marban, 2020). The high inflammatory burden precipitated by COVID-19 worsens the condition of patients with pre-existing cardiovascular disease and provokes myocardial damage (Shi et al., 2020). COVID-19 can induce new cardiac pathologies or aggravate pre-existing cardiovascular diseases, resulting in significant cardiac sequelae (Pranata et al., 2020). Of 416 patients hospitalized in China, 19.7% had resultant cardiac injury (Shi et al., 2020). When this group was compared to those without cardiac injury, it was established that patients were older (median age of 74 years) and had more comorbidities including chronic heart failure, diabetes, hypertension, and coronary heart disease (Shi et al., 2020). Cardiac involvement has become a prominent feature of COVID-19 and is likely to be of prognostic value.

### Renal Manifestations

The prevalence of acute renal impairment with COVID-19 infection is frequent, reported in the range of 0.5%–36.6% (Yang et al., 2020; Hirsch et al., 2020). However, early signs of kidney dysfunction manifesting with elevated levels of blood urea nitrogen (BUN) and serum creatinine, proteinuria, and haematuria have been reported at much higher rates (Gupta et al., 2020). In one New York based study, it was found that 87% of patients had proteinuria (Cummings et al., 2020), while 40.9% were found to have haematuria in a separate study (Hirsch et al., 2020). The renal tropism of SARS-CoV-2 is thought to be related to the increased number of ACE-2 receptors expressed in the brush border of the proximal tubular cells (Benedetti et al., 2020). AKI has been positively correlated with disease severity and prognosis, with higher rates of mortality reported among those with an AKI. Moreover, those with greater renal impairment were found to have more coexisting conditions; therefore, this represents a risk factor especially in older adults (Puelles et al., 2020).

### Thromboembolic Events

As well as the aforementioned biological complications of COVID-19 infection, systemic coagulopathy is common in severe cases of infection. While the lungs are the primary target for COVID-19, inducing ARDS, an associated thrombo-inflammatory state has been described (Ribes et al., 2020). The thrombogenicity of this infection has been demonstrated by the high incidence of associated thrombotic events and abnormal haemostasis parameters, despite treatment with anticoagulation (Helms et al., 2020b). It has been seen that patients with more severe disease are prone to developing pulmonary embolisms, deep vein thrombosis, and arterial thrombosis, which is further complicated by disseminated intravascular coagulopathy (DIC) (Di Minno et al., 2020). The high rate of residual chronic disability among older adults makes adequate screening procedures and antithrombotic strategies essential to patient management to avoid the possibility of protracted and potentially fatal outcomes within this cohort.

### Neurological Manifestations

The neurological complications of COVID-19 and the prevalence of these complications are still not fully known. A study conducted in Wuhan, China documented that 36.4% of COVID-19 positive patients experienced neurological symptoms, with neurological involvement being more prominent in patients experiencing a severe infection. The most common symptoms included dizziness, headache, and olfactory and gustatory dysfunction (Mao et al., 2020). This was supported by a United Kingdom (UK)-wide surveillance study where 62% of patients experienced a cerebrovascular event, with stroke being the most common presentation. Those presenting with a cerebrovascular event were mainly over 60 years old, while altered mental status was seen almost equally in all age groups. Furthermore, 59% of patients fulfil the criteria for a psychiatric diagnosis (Varatharaj et al., 2020).

There is evidence to suggest that COVID-19 can produce long-term neurological effects by either eliciting a new disorder or acting on an underlying neurological condition. This has been supported by findings that 33% of patients continued to experience some decrease in cognitive and motor function after they were discharged (Helms et al., 2020a). It is also believed that the cytokine storm and the systemic inflammation that follow COVID-19 infection can lead to the formation of new cognitive impairment (Iwashyna et al., 2010) and neurodegeneration (Cunningham, 2012).

## Psychological

It has long been recognized that patient health and well-being does not return to baseline immediately after resolution of critical illness and discharge from hospital. In fact, ‘post intensive care syndrome’ (PICS) is a term developed by the Society of Critical Care Medicine to describe the symptoms many experience following recovery from a severe illness requiring intentive care unit (ICU) admission, which may persist for years (Elliott et al., 2014). Included in this syndrome are declines in both cognitive function and mental health. Following ARDS, psychiatric disorders such as post-traumatic stress disorder (PTSD), depression, and generalized anxiety have all been identified (Herridge et al., 2016). PICS can interfere with patient outcomes following recovery from viral illness, and patients at risk must, therefore, be identified and provided with psychological intervention in a timely and appropriate manner.

### Mental Health in Older Adults With Comorbidities

Older adults, especially those with pre-existing cognitive impairment and those who are socially isolated, may disproportionately experience the negative psychiatric effects of PICS (Lloyd-Sherlock et al., 2020; Sheffler et al., 2020). For example, older adults appear to be the most severely affected by the diagnosis of PTSD that ensues in 30% of patients following ICU admission for ARDS (Herridge et al., 2003). Coupled with the finding of alarmingly high rates of suicide worldwide in this population (Sheffler et al., 2020), this suggests that it is important to closely monitor the older adult population following ICU admission. Many explanations for high rates of suicide in this age group have been proposed, including the simultaneous experience of ‘thwarted belongingness’ and ‘perceived burdensomeness’ associated with a decline in health in older populations (Cukrowicz et al., 2011; Sheffler et al., 2020). This is especially relevant in the context of the COVID-19 pandemic during which older adults are already isolating themselves from family members and the public. Furthermore, those with COVID-19 must socially isolate and become completely reliant on the care of others (healthcare workers, family members, or other social supports) for the duration of their illness, which, in the case of ARDS, may be prolonged and physically debilitating.

### Psychological Effects Post SARS Infection

Consistent with above, a heightened prevalence of psychiatric disorders (including depression and PTSD) was identified in patients following recovery from the 2002 SARS epidemic via the SARS virus (Mak et al., 2009). Coinciding with the SARS outbreak was also a specific increase in suicide-related deaths among individuals over 65, which has been attributed to increased fear of becoming ill, burdening family members, and social disconnection (Yip et al., 2010).

### Early Psychological Effects Post COVID-19

In the short term following the acute illness, COVID-19 survivors have been found to screen positive for anxiety, depression, and PTSD, resulting in decreased quality of life (Mendez et al., 2020). This is especially true for those with pre-existing stress-related symptoms, as is described in many older adult populations, and those with substance abuse disorders, who experience reduced access to support systems (Satre et al., 2020). Over one-third of older adults surveyed have identified experiencing symptoms of anxiety and depression since the beginning of the COVID-19 pandemic (Meng et al., 2020), unfortunately coinciding with reduced access to psychiatric care during the same time period (Van Dorn et al., 2020). In contrast to expected outcomes, survivors of COVID-19 above the age of 60 have reported reduced symptoms of both depression and anxiety compared to their younger counterparts (Xin et al., 2020), as well as stable mental health despite increased feelings of loneliness (Van Tilburg et al., 2020). This suggests heightened resiliency of the older adult population in the face of the current pandemic. For those who experienced depression, loneliness was found to be the mediating factor in those who had strong interpersonal connections, whereas those who felt disconnected from social networks had higher rates of depression regardless of loneliness (Krendl & Perry, 2020). More long-term studies regarding psychological well-being in this population of COVID-19 survivors are not yet available, but may resemble those conducted following the 2002 SARS epidemic.

### Molecular Mechanisms of Mental Health Decline Post COVID-19

Many mechanisms underlying the psychiatric sequelae following COVID-19 infection have been proposed, involving immune dysfunction, pharmacotherapy, and the gut–brain axis (Troyer et al., 2020). Aberrant immune processes may be implicated in the development of psychiatric dysfunction following recovery from COVID-19. One theory outlines the involvement of neuroinflammation induced by virally infected myeloid cells and constitutively active lymphoid cells, resulting in neuroinflammation that outlasts the acute infection. Alternatively, lymphocytes that respond to COVID-19 epitopes may cross-react with human myelin in those who are vulnerable in a process termed molecular mimicry. Both processes may result in prolonged neuropsychiatric complications in the survivor. Immunomodulatory corticosteroid therapy can be given to COVID-19 patients who experience a hyperinflammatory response during acute infection. These agents are known to cause various psychiatric side effects, including disturbances in both cognition and sleep, delirium, mania, depression, and psychosis. It has also been proposed that COVID-19 infection may induce alterations in patient microbial composition, which may in turn affect the gut–brain axis resulting in the manifestation of neuropsychiatric symptoms (Troyer et al., 2020).

## Social

### Loneliness and Social Isolation

We are in the midst of a dual pandemic: COVID-19 and loneliness/social isolation. As articulated above, older adults are disproportionately impacted by COVID-19 and are at an increased risk of both morbidity and mortality from the virus (Wang et al., 2020; Nikolich-Zugich et al., 2020). Older adults are also at an increased risk of experiencing both loneliness and social isolation (Centre for Disease Control and Prevention, 2020a). Consequently, the intersection of these two pandemics has compounding health effects.

While used interchangeably, loneliness and social isolation are separate entities (Brooke & Jackson, 2020). Loneliness is a subjective feeling or emotion often associated with a lack of connectedness. Social isolation is a lack of contact with social circles and networks as a result of restrictions that have been put in place. Both can exert negative health outcomes (Hwang et al., 2020).

Prior to the COVID-19 pandemic, globally there was wide recognition that loneliness and social isolation were endemic to society. In 2017, US Surgeon General Vivek Murthy called loneliness a ‘global epidemic’ amongst older adults worldwide (Berg-Weger & Morley, 2020), and in 2018, the UK appointed a Minister of Loneliness and deemed loneliness to be ‘the greatest public health challenge of our time’ (Prime Minister’s Office, 2018; BBC News, 2018). The risk associated with a lack of social connection is considered to be comparable to smoking 15 cigarettes a day (Holt-Lunstad, 2017; Hwang et al., 2020), and has been linked to an increased risk of heart disease, anxiety, depression, cognitive decline, Alzheimer’s disease, and death (Cacioppo & Cacioppo, 2014). Conversely, meaningful social engagement has a protective effect on health (Cacioppo et al., 2015).

Globally, in order to curtail the virus and to keep people safe, public health measures such as quarantine, restricting movements, self-isolation, social distancing, and ‘cocooning’ were introduced (Ward et al., 2020). Given the increased risk of severe disease amongst people with comorbidities and those over the age of 60, these measures have been more limiting to these groups (Shahid et al., 2020). This has led to the adoption of a conservative public health approach in many countries with an added emphasis placed on older adults to stay home (Brooke & Jackson, 2020). This response, while essential to protect older adults from exposure to the virus, has led to increased loneliness, isolation, and negative stereotyping of older adults (Monahan et al., 2020; The Lancet Infectious Disease, 2020).

Research suggests that COVID-19 may exacerbate the feelings of isolation and loneliness for people who reported feeling this way prior to the pandemic (Brooke & Jackson, 2020). For those who did not report feeling isolated or lonely prior to the pandemic, there is a concern that they may be disproportionately affected because social networks and outings, such as grocery shopping, attending places of worship, and community programming, may have been suddenly discontinued. Research from Hong Kong that collected data from close to 600 adults over the age of 60 reported a significant increase in loneliness since the pandemic started (Wong et al., 2020). A Dutch study of close to 2000 people 65 years and older found a large increase in emotional loneliness–absence of close emotional attachment–during the pandemic (Van Tilburg et al., 2020).

### Income and Job Insecurity

In response to the escalating spread of COVID-19, countries around the globe have adopted lockdown measures which have decreased global trade and jeopardized innumerable jobs.

Income and employment are two of the most important determinants of health (Heymann, 2006; Benach et al., 2014). Older adults, especially the millions who live without the support of a pension, are most physically and financially vulnerable to COVID-19. Since the onset of the pandemic, there has been a purported 7% rise in early retirement (Furceri et al., 2020). As such, older adults are left with decreased financial security and reduced lifetime earnings. The rationale behind the early retirements is unclear, but it is hypothesized to be a combination of health and safety concerns in the work environment, an inability to telework, or ageism. Unplanned retirement and job loss prior to retirement have been shown to increase the likelihood of anxiety and depression experienced by older adults (Gallo et al., 2006).

The societal move to digital communication has also resulted in older adults becoming increasingly disconnected from society, unable to navigate social supports such as search engines or social/video networking sites (Micheli et al., 2019). During a time where Zoom calls and video chats are part of our everyday vocabulary, limited access to technology or an unfamiliarity with technology not only contributes to social isolation and loneliness but also makes working from home difficult for older adults that have not yet retired. According to Anderson and Perrin (2017), 49% of adults aged 65 and older do not have internet access and are significantly less likely to own smartphones compared with adults aged 18 to 64.

According to Blewett et al. (2019), older workers constitute a larger proportion of the frontline workforce than the workforce as a whole. Pre-pandemic employment data from the US show that men above the age of 62 are most commonly employed in manual labour jobs (e.g. transport, farming, and janitorial services), making it impossible to transition to working from home (Johnson & Wang, 2017). Moreover, these frontline workers are often financially unprotected (earning minimum wage without healthcare or paid sick leave) and in close contact with potentially infected individuals. During the outset of the pandemic, widespread personal protective equipment (PPE) shortages and absent social distancing protocols left millions of frontline workers without basic protection. Older adults fearing viral exposure were also left without social protection (i.e. unemployment insurance benefits) if they chose to resign from their jobs (Guasti, 2020).

### Food Insecurity

Even before the COVID-19 pandemic, older adults were particularly vulnerable to food insecurity due to income-based and accessibility barriers, as well as cognitive decline and social isolation (Vilar-Compte et al., 2017). The pandemic has only served to further impede the ability of older adults to obtain healthy food. For example, the global lockdown measures have prevented older adults from accessing nutrition programs, and supermarket trips are impeded due to fear of viral acquisition or inadequate financial means. The pandemic also created uncertainty in the global food chain and led to instances of hoarding or panic buying wherein those who could afford to stockpile food, supplies, and water created market shortages. These shortages led to price inflation and left high risk populations, such as older adults, food insecure (Lee, 2020).

The prevalence of food deserts in rural areas also makes older adults in such areas increasingly vulnerable. When grocery stores do exist, they are less likely to have the infrastructure in place for grocery delivery (Kusch-Brandt, 2020).

## Conclusion

The biopsychosocial model of COVID-19 highlights the complex interactions between the physical, psychological, and social underpinnings of the virus (Wong et al., 2020). Each cannot be siloed as their interconnectedness is pivotal for the development of supportive interventions and rehabilitation.

The impact of COVID-19 on health after infection can be recognized in multiple biological systems. For example, patients with pre-existing cardiovascular disease have experienced worsening of their condition. Additionally, kidney dysfunction may occur, as well as olfactory and gustatory dysfunction. Furthermore, COVID-19 has been implicated in declines in cognitive and motor function, as well as the development of new psychiatric diagnoses.

Data from prior epidemics (SARS) and limited data from the current COVID-19 pandemic indicate that psychological sequelae are prevalent in survivor populations, especially older adults. However, the psychological resiliency of this population has also been demonstrated. Many biological mechanisms have been suggested to underpin the psychiatric phenomena that occur following recovery from infection.

Social distancing and stay at home measures, interventions aimed at protecting vulnerable populations, are paradoxically leaving older adults more isolated and food insecure secondary to the COVID-19 pandemic. Furthermore, a disproportionate number of older adults work in either at-risk jobs (that are most susceptible to shutdowns during the global lockdown) or essential services where the risk of exposure is highest. This results in older adults facing intertwined financial and health risks due to the pandemic and the resulting economic shutdown.

An awareness of the biological, psychological, and social sequelae of a COVID-19 infection is of major importance for the development of interventions, preventions, and rehabilitation for older adults.
